# Landscape of alterations in the checkpoint system in myelodysplastic syndrome and implications for prognosis

**DOI:** 10.1371/journal.pone.0275399

**Published:** 2022-10-25

**Authors:** Ivan Moiseev, Nikolai Tcvetkov, Olga Epifanovskaya, Elena Babenko, Anna Parfenenkova, Evgenii Bakin, Ksenia Yurovskaya, Elena Morozova

**Affiliations:** RM Gorbacheva Research Institute, Pavlov University, Saint-Petersburg, Russian Federation; Stellenbosch University Faculty of Medicine and Health Sciences, SOUTH AFRICA

## Abstract

The emergence of novel immunotherapies for myelodysplastic syndrome (MDS) calls for a profound characterization of the "immunome" in the bone marrow (BM) and evaluation of prognostic impact of immunological changes. We performed a prospective study of 87 MDS patients who were referred to a tertiary hematological center and of 11 bone marrow donors who were not related to the study cohort. A flow cytometry panel with 48 markers including checkpoint ligands and receptors was used to study lymphoid and myeloid subpopulations in the bone marrow aspirates. The study found that both the healthy donors and the MDS patients have a high proportion of lymphocytes with PD-1 expression (41±18% and 58±25% respectively) and a high proportion of myeloid cells with PD-1L expression (31±23% and 12±7% respectively), indicating a potential physiological role of checkpoint systems in BM. At the same time, complex alterations including PD-1, CTLA-4, LAG-3 and TIM3 pathways accompanied by an increased level of T-reg and myeloid derived suppressor cell populations were identified in the BM of MDS patients. Cluster analysis showed independent prognostic significance of the checkpoint profile for overall survival (HR 1.90, 95%CI 1.01–3.56, p = 0.0471). TIM3-postive NK and CD8 effector cells along with the blast count were the key subpopulations for prognosis. An elevation of blasts in the bone marrow was associated with simultaneous expression of multiple checkpoints on myeloid cells.

## Introduction

Myelodysplastic syndrome (MDS) is a heterogenic group of diseases characterized by accumulation of somatic mutations in hematopoietic stem cells [1–3] and bone marrow niche abnormalities [4] which lead to ineffective hematopoiesis, cytopenia and possible transformation into acute myeloid leukemia [5]. Significant progress was made recently in understanding how somatic mutations impact the clinical course of this disease. Prognostic models are currently being refined to incorporate genetic features [6]. Nonetheless, it is possible that genetic alterations are not the sole determining factor for the prognosis. It was demonstrated that immune-mediated cell death [7, 8] and significant alterations in the checkpoint system [9, 10] occur throughout the course of the disease. Because of these features Winter and co-authors recently proposed studying the "immunome" along with the genetic aberrations in MDS patients [11].

Clinical evaluation of PD-1 and CTLA4 inhibitors in MDS patients demonstrated moderate efficacy with predominantly stable disease as best response [12, 13]. However subsequent studies, including a study by our group [14], demonstrated complex alterations in checkpoint system including TIM3-Galectin axis [15], PD-1 axis [16], LAG3 [17] and CD47 [18]. Promising results were seen for checkpoint inhibitors targeting CD47 [19] and TIM3 [20], and thus they can be considered possible effective immunotherapies for MDS. These findings show that further profound characterization of the changes in the "checkpoint" system both in myeloid cells and lymphocyte subpopulations is needed. This will give a better understanding of the characteristics of MDS "immunome" and allow for using the novel treatment tools accordingly. It is for these reasons that we conducted a prospective study to extensively evaluate expression of checkpoints on various subpopulations in bone marrow of MDS patients and compared the results to those of healthy bone marrow donors.

## Methods

### Study population

87 consecutive patients with confirmed diagnosis of MDS were enrolled in the prospective study. All the patients were referred to the tertiary hematological center, Raisa Gorbacheva Memorial Research Institute of Children Oncology, Hematology and Transplantation at Pavlov University, and underwent bone marrow aspiration during diagnostic workout. All the patients had unequivocal laboratory data for MDS both at Pavlov University and the referring centers. The enrollment target was 80 patients with 10 additional patients in case some of the collected bone marrow specimens were of inadequate quality. Eleven bone marrow donors without granulocyte colony stimulating factor priming donating to patients outside of the study cohort were also enrolled in the study. Enrollment period lasted from 2016 to 2021. Written informed consent was obtained from all the patients and donors to use their biological material and personal data for research purposes. The study was approved by the Ethical committee of the First Pavlov Medical University and performed in accordance with the ethical standards laid down in the 1964 Declaration of Helsinki and its later amendments.

The median age of the patients was 48 years (range 18–74). Patient and disease characteristics are presented in Table 1. All the patients with excess blasts were treated with hypomethylating agents (5-azacitidin or decitabine) and 20 patients underwent subsequent hematopoietic stem cell transplantation. Low risk patients were treated with erythropoietin or luspatercept. Patients with isolated del 5q were treated with lenalidomide.

**Table 1:** Patient and disease characteristics.

Parameter	N (%)
Gender	
Male	50 (57%)
Female	37 (43%)
Diagnosis	
MDS with single lineage dysplasia	10 (11%)
MDS with multilineage dysplasia	22 (26%)
MDS with excess blasts I	16 (18%)
MDS with excess blasts II	37 (43%)
MDS with isolated del 5q	2 (2%)
IPSS-R	
Very low	2 (2%)
Low	18 (21%)
Intermediate	17 (20%)
High	22 (25%)
Very high	28 (32%)
Karyotype	
Normal	43 (48%)
Del 5q	5 (6%)
Trisomy 8	9 (10%)
Monosomy 7	6 (7%)
Complex, monosomal	11 (13%)
Complex, non-monosomal	4 (5%)
Translocations of chromosome 3	5 (6%)
Other	4 (5%)

### Flow cytometry

The fresh non-frozen samples of bone marrow aspirate were studied by flow cytometry (FACS Canto II, BD Biosciences, CA, USA; antibodies by Miltenyi biotec, Bergisch Gladbach, Germany). At least 300 thousand events were collected. The following surface markers were used to identify lymphocyte, regulatory and myeloid subpopulations: CD45, CD3, CD4, CD8, CD16, CD56, CD25, CD127, HLA-DR, CD117, CD34, CD15, CD11b, CD127, CD25, CD4. The list of checkpoint receptors evaluated on lymphocyte subpopulations included CD279 (PD-1), CD152 (CTLA-4), CD278 (ICOS), CD223 (LAG-3), TIM3, CD272 (CD272). The following checkpoint ligands were evaluated on myeloid cells and regulatory subpopulations: CD274 (PD-1L), CD273 (PD-1LG2), CD275 (B7-H2, ICOS ligand), CD276 (B7-H3) and CD80 (B7-1) and CD86 (B7-2), galectin-9. The disposition of antibodies and fluorochromes is presented in S1 Table. The list and the description of subpopulations analyzed is available in S2 Table.

### Statistical analysis and definitions

The four-year overall survival (OS) was calculated using the Kaplan-Meier method from the time of diagnosis to the time of death. The median follow-up was 18 months (range 2–60 months). Multivariate analysis was performed using the technique of Cox regression. Patients who underwent allogeneic stem cell transplantation were censored at the time of transplantation.

Data was analyzed as the percentage of nucleated cells. Additional visualization of differences for healthy volunteers and MDS patients was performed based on the percentage of the cell expressing checkpoint receptors or ligands. Comparison of healthy volunteers and patients, as well as patients who died and who survived during the follow-up was done in several steps. Univariate logistic regression screening was performed for the initial feature extraction. Cluster analysis was performed for the visualization of patterns in cell subpopulations. The identified differences between the clusters were presented as mean±SD, the p-values were produced by Wilcoxon test only for representative purposes. Due to the preceding steps of cluster and principal component analysis no adjustment of p-values to account for the multiple comparisons issue was applied. Principal component analysis (PCA) was used to identify patterns in bone marrow composition in relation to survival in patients [21]. Based on the PCA results cluster extraction analysis was performed. The identified clusters were further characterized and evaluated against the donor and OS status [22]. Spearman correlation was used for analyzing the interaction between cells subpopulation in patients. Bonferroni correction was used in correlation analysis for multiple comparisons. The findings were visualized by building correlation matrixes with correlation p-values presented as a heat-map plot. Data processing and visualization was done using the R statistical packages and SAS 9.4.

## Results

### Profile of checkpoint receptors in patients and healthy donors

**Fig 1:**
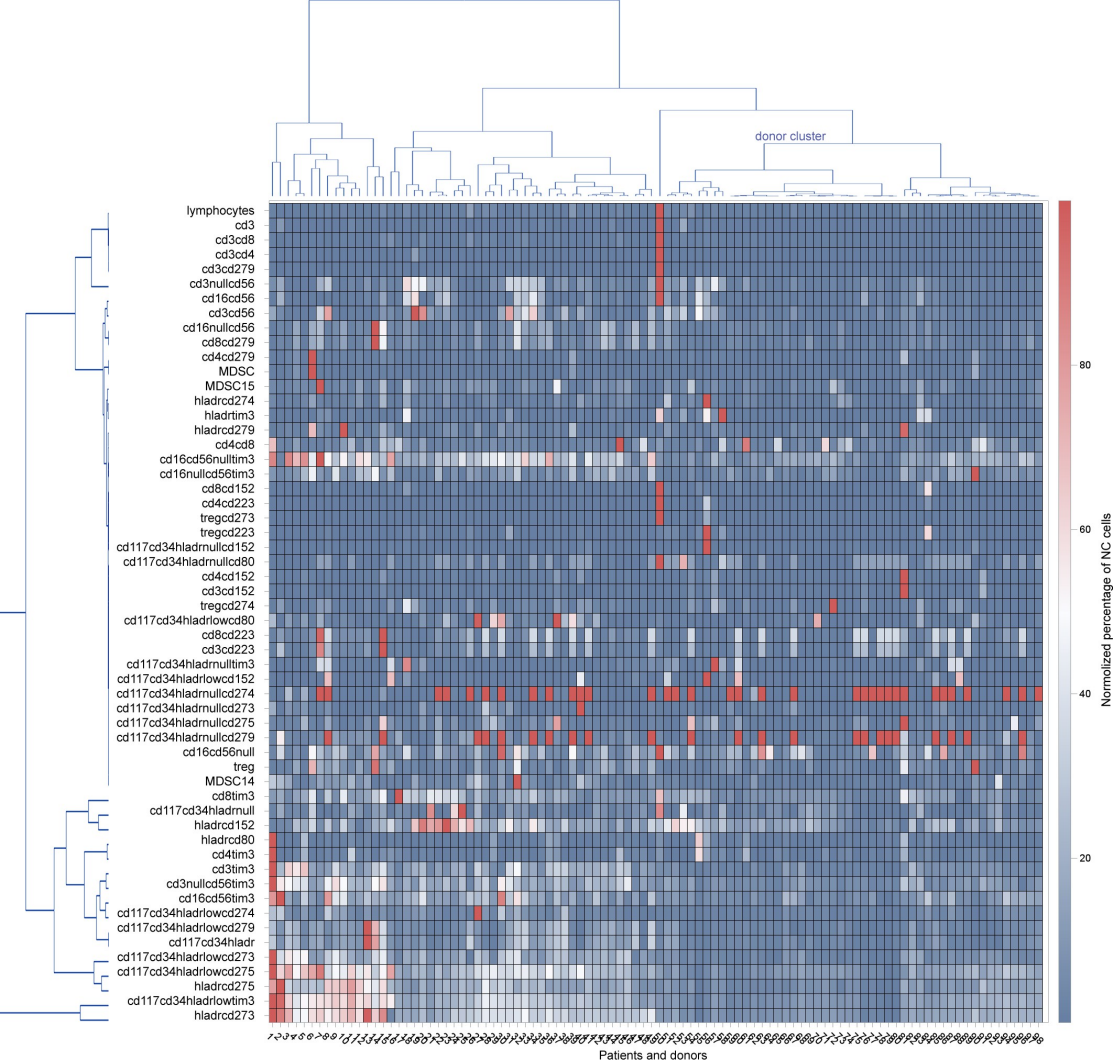
Cluster analysis and heatmap of normalized percentages of cell populations in bone marrow. Red colors represent higher percentages within the study group, blue colors represent lower percentages and white colors represent median percentages in individual patients and donors.

Complex alterations in bone marrow composition were observed in MDS patients compared to healthy volunteers. In cluster analysis 9/11 donors fell into distinct cluster that was characterized by a reduced number of lymphocytes and their subsets, reduced number of myeloid-derived suppressor cells (MDSC) and T-regulatory cells (T-reg) compared to another clusters (Fig 1). More specifically, MDS patients had a significantly higher prevalence of all common lymphocyte subpopulations (CD3, CD4, CD8, NK cells p<0.05). The difference in NK cell prevalence was due to higher numbers of CD16+CD56+ (p = 0.0143) and CD16-CD56+ (p = 0.0019) subpopulations, but not CD16+CD56- (p = 0.32). No difference in prevalence of NKT cells was observed (p = 0.08). MDS patients had a significantly increased percentage of T-regulatory (T-reg) cells (0.82±0.63% vs 0.45±0.17%, p = 0.0344) and CD15-positive myeloid-derived suppressor cells (MDSC) (2,17±5,15% vs 0,21±0,15%, p = 0.0007). On the contrary the level of CD14-positive MDSC was not increased in MDS patients (S3 Table).

The profile of checkpoint receptors was also altered in MDS patients. They had a significantly increased proportion of PD-1-positive CD3 (p = 0.0007), CD8 (p = 0.0024) and CD4 cells (p = 0.0025), CTLA-4-positive CD3 (p = 0.0057), CD8 (p = 0.0415) and CD4 cells (p = 0.0102), LAG-3-postive CD3 (p = 0.0022), CD8 (p = 0.0128) and CD4 cells (p = 0.0324), TIM3-positive CD3 (p <0.0001), CD8 (p = 0.0029) and CD4 cells (p = 0.0005). The percentage of TIM-3 positive NK cells was increased in MDS patients and this was true for all NK subpopulations: CD16+CD56- (p = 0.0019), CD16-CD56+ (p = 0.0121), CD16+CD56+ (p = 0.0066) (S3 Table).

The analysis of fractions of checkpoint receptors expressing cells confirmed the differences in PD-1 (p = 0.0013), LAG3 (p = 0.0167), TIM3 (p = 0.0047) expression on T-cells. Interestingly, both in healthy donors (41±18%) and MDS patients (58±25%) we detected a high proportion of PD-1 positive T-cells and high PD-1 expression was confirmed both on CD4+ and CD8+ fractions. In MDS patients TIM3 was also expressed on a higher proportion of T-cells compared to healthy donors (3.95±9.51% vs 0,62±0,31%). The highest percentage of TIM-3 positive cells was observed on NK subpopulations both in MDS patients and in healthy volunteers: 33,01±23,89% vs 11,60±9,98% of CD16+CD56- NK cells, 13,16±18,80% vs 14,86±28,80% of CD16-CD56+ NK cells and 45,55±27,63% vs 28,17±24,30% of CD16+CD56+ NK cells respectively (S4 Table).

### Profile of checkpoint ligands in patients and healthy donors

Due to presence of excess of blast forms in MDS the number of myeloid precursors, both CD117+CD34+HLA-DRlow (p = 0.0241) and CD117+CD34+HLA-DRneg (p = 0.0238), was higher in MDS patients than in healthy donors. Thus only T-reg and MDSC subpopulations were analyzed as the percentage of total cells. There was a significantly higher number of PD-1L-postive (p = 0.0308), PD-1LG2-positive and LAG-3-positive (p = 0.0221) T-regs in MDS patients (p = 0.0092). However absolute levels of these cells were very low, comprising less than 0.05% of all nucleated cells. The level of PD-1L and PD-1LG2 positive MDSC was not increased in MDS patients despite their higher overall levels (S5 Table).

The analysis of fraction of hematopoietic precursors expressing checkpoint ligands demonstrated that a significantly higher fraction of CD117+CD34+HLA-DRlow in MDS patients carried PD-1L (31,06±23,28% vs 12,04±7,48%, p = 0.0093). It was also confirmed that a higher fraction of Tregs carried PD-1L (p = 0.0462) and PD-1LG2 (p = 0.0394) ligands in MDS patients, but the percentage of such cells was low: 5,19±12,00% and 0,45%±0,74% respectively. The prevalence of checkpoint ligand expression on MDSCs was comparable between MDS patients and healthy donors (S6 Table).

**Fig 2:**
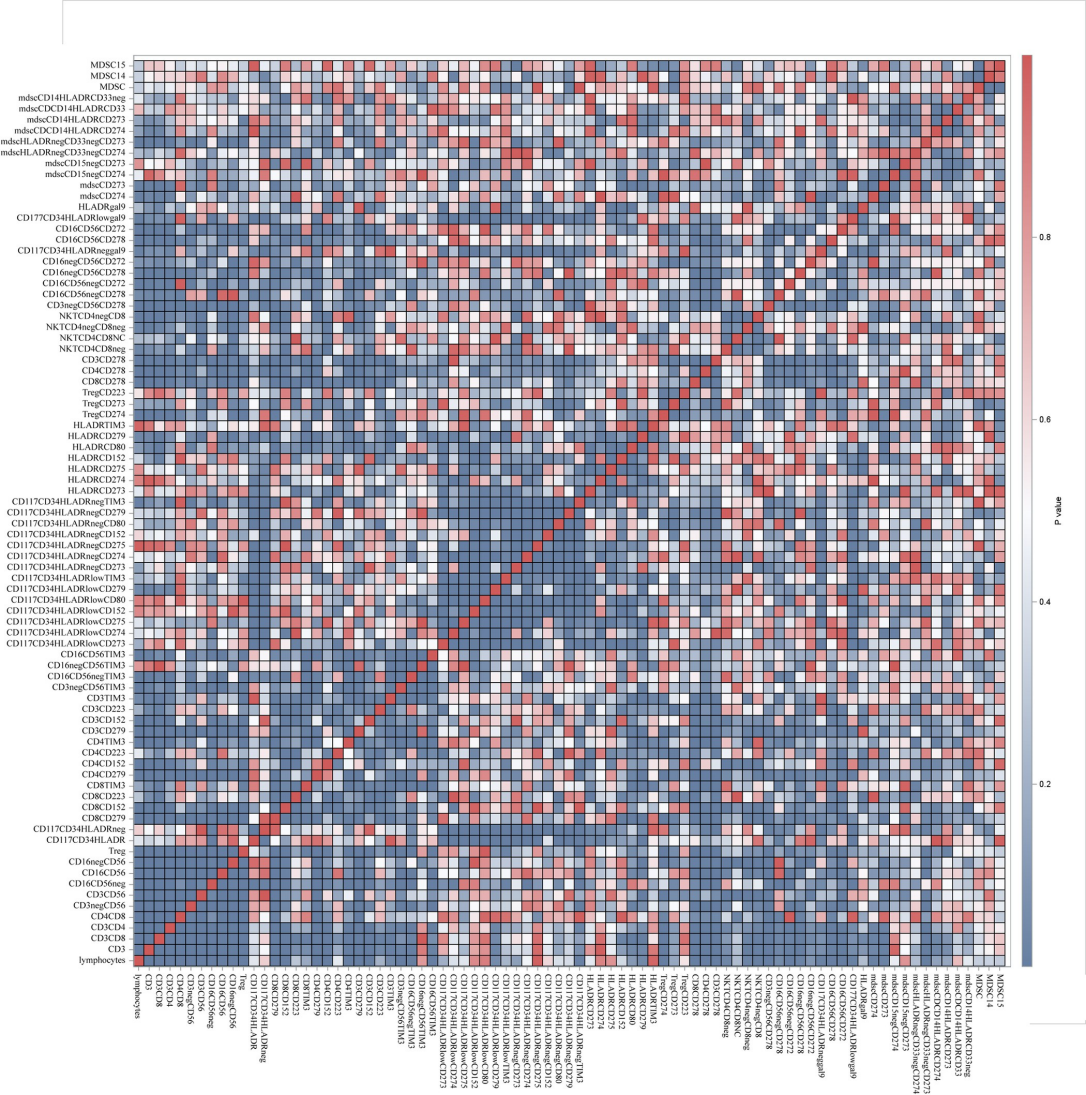
Correlation matrix with statistical significance. P-values are transformed into colors. Dark blue colors represent significant correlations. All other colors–non-significant.

The correlation analysis incorporating only MDS patients showed strong positive association between the level of PD-1L positive blast cells, the overall level of CD117+CD34+HLA-DRlow blasts (r = 0.80, p<0.0001), PD-1LG2 blasts (r = 0.43, p<0.0001), blasts with ICOS ligand (r = 0.44, p<0.0001), blasts with PD-1 receptor (r = 0.53, p<0.0001), overall pool of HLA-DR cells with PD-1L expression (r = 0.38, p = 0.0003), galectin-9-postive blasts (r = 0.53, p<0.0001). Surprisingly, no significant correlation was observed with any of the lymphocyte subpopulations (Fig 2). The level of Treg significantly correlated with the overall prevalence of CD3 (r = 0.88, p<0.0001), CD4 (r = 0.88, p<0.0001), CD8 (r = 0.88, p<0.0001), NKT (r = 0.43, p<0.0001), all subpopulations of NK cells (r = 0.51–0.53, p<0.0001), level of PD-1-positive CD8 (r = 0.62, p<0.0001) and CD4 cells (r = 0.65, p<0.0001), level of CTLA-4-positive CD8 (r = 0.38, p = 0.04), level of TIM3-positive CD8 (r = 0.62, p<0.0001) and CD4 cells (r = 0.65, p<0.0001), CD16+CD56- NK cells (r = 0.38, p<0.0001), ICOS-positive CD4 (r = 0.54, p<0.0001) and CD8 cells (r = 0.55, p<0.0001). No significant correlations were identified for MDSC.

### Prognostic significance of checkpoint profile

**Fig 3:**
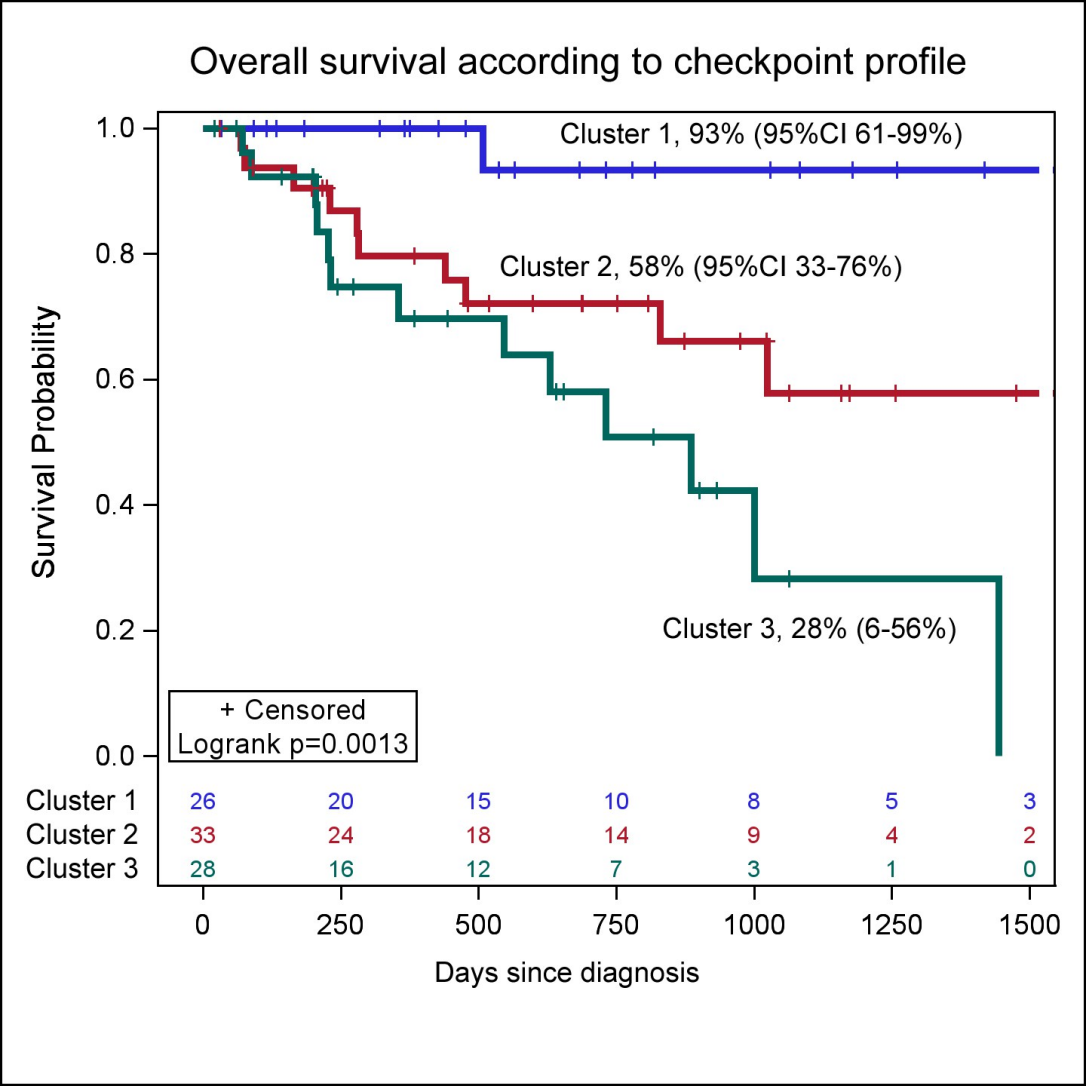
Overall survival probability according to clusters with different checkpoint profiles.

Principle component analysis identified only several major drivers of mortality. Negative impact was observed for a higher percentage of CD117+CD34+HLA-DRlow blast cells, higher percentage of TIM-3-positive CD8 cells and CD16-CD56+ NK cells. Positive impact was observed with high prevalence of CD16-CD56+ expressing ICOS. Based on PCA clusterization, three major clusters were identified. Cluster 1 was characterized by low expression of TIM3 on CD3+ lymphocytes (p = 0.0105), NK cells (p < .0001) and lower prevalence of PD-1-positive (p = 0.0045) and PD-1L-postive myeloid cells (p = 0.0064). Cluster 2 was characterized by a higher prevalence of CD8+TIM3+ cells (p = 0.0030), CD4+TIM3+ cells (p = 0.0204), CD56+TIM3+ NK cells (<0.0001) (S7 Table). Cluster 3 was characterized by a higher prevalence of blasts (p<0.0001), PD-1-positive (p = 0.0053), PD-1L (p<0.0001), PD-1L G2(p<0.0001), TIM3-postive (p = 0.0013) and CD80-positive (p<0.0001) blasts (S7 Table). Patients in Cluster 1 had significantly better survival (93%, 95%CI 61–99%) than in Cluster 2 (58%, 95%CI 33–76%). Worse survival was observed in patients from Cluster 3 (28%, 6–56%) (Fig 3). When corrected for the clinical risk parameters with IPPS-R score (HR 2.07, 95%CI 1.18–3.61, p = 0.0108), the identified clusters retained independent prognostic significance (HR 1.90, 95%CI 1.01–3.56, p = 0.0471, S1 Fig).

## Discussion

To our knowledge this is one of the largest studies with a complex analysis of checkpoint alterations in MDS patients and healthy volunteers. Since the novel immunotherapeutic methods are already used in clinical studies it was not unexpected that we would detect some significant changes in a checkpoint profile. However, the presence of large PD1-positive T-cell populations in healthy bone marrow was an interesting finding. A shift in paradigm of B- and T-cell role in hematopoiesis has occurred recently. It was previously believed that the memory B- and T-cells resided in a bone marrow niche and were protected from apoptosis [23, 24]. Now, a concept of an active role of these cell populations has emerged. It was demonstrated that T-cells support inflammatory and probably steady state hematopoiesis through secretion of interferon-γ, IL-6, CXCR4, IL-17A, IL-1 and G-CSF. The effects of these cytokines are mediated either directly through stem cells or through endothelium and mesenchymal cells [25–27]. Therefore, the observed PD1-positive subset of T-cells in healthy persons might represent this regulatory population.

In fact, we demonstrated that a number of checkpoint ligands were also overexpressed on myeloid cells in healthy bone marrow. The previous studies were predominantly focused on patients with malignant disease and considered the expression of checkpoint ligands on myeloid cells in the frame of paraneoplastic syndrome [10, 28, 29]. But our data indicates that ligand expression may have physiological nature and represent another mechanism of hematopoiesis regulation. The increment of checkpoint ligand expression might as well be a response to stress and inflammatory conditions or can be driven by somatic mutations and represent a tumor resistance mechanism. These pathogenic features need further elucidation.

Another important observation in this study was a significant correlation between the expression of checkpoint receptors and ligands on myeloid cells in MDS patients. It was demonstrated that the expression of both checkpoint ligands and receptors, including PD-1, PD-1L and TIM3, CTLA-4 and some others was regulated through similar signaling pathways, i.e. JAK, STAT, MEK, MAPK, PI3K, AKT [30–33]. Given the fact, that PD-1L expression in MDS is altered after treatment with hypomethylating agents [10], it is likely that epigenetic mechanisms are involved in this complex deregulation. The complexity of alterations may to some extent explain moderate activity of monoclonal antibodies against PD-1, CTLA-4 and LAG-3 in MDS [12, 13]. Surprisingly, these complex alterations had limited separate predictive power over clinical features of a high-risk disease, like the blast count. The only important subpopulations for prognosis were TIM-3 and ICOS expressing NK cells and to some extent TIM-3-expressing CD8 cells. It is known that NK cell activity is important for disease control in MDS and acute myeloid leukemia [34]. A growing body of evidence indicate that NK cells is a heterogenic population with some degree of plasticity [35]. Our study confirmed that NK cells were also the target of checkpoint regulation and a compartment of NK cells should be monitored closely during treatment with novel immunotherapies.

The major limitation of the study is an absence of certain important checkpoints in the study panel. Since the beginning of the study, it was shown that overexpression of TIGIT on NK and T cells facilitated immune escape in MDS [36]. It was also shown that CD47/SIRPα is an important axis in MDS [18]. These two checkpoints were not evaluated in our study. However, from a relatively large panel of checkpoints we identified the leading role of TIM3 and PD-1 with limited involvement of other signaling pathways. Future studies can focus on incorporating these two pathways into treatment strategies. The validation of NK cell checkpoint status as the easy marker of MDS "immunome" will require further multicenter validation.

In conclusion, we demonstrated an important role of checkpoints in normal and malignant hematopoiesis. Incorporation of immunological markers into prognosis systems will require further validation.

## Supporting information

S1 Table Disposition of antibodies and fluorochromes. (PDF)

S2 Table List of subpopulations analyzed. (PDF)

S3 Table Percentage of subpopulations with checkpoint receptors from total nucleated cells in bone marrow in healthy donors and MDS patients. Differences determined in cluster analysis are discussed in the text.
(PDF)

S4 Table Percentage of cells expressing checkpoint receptors in healthy donors and MDS patients. (PDF)

S5 Table Percentage of subpopulations with checkpoint ligands from total nucleated cells in bone marrow in healthy donors and MDS patients. Differences determined in cluster analysis are discussed in the text.
(PDF)

S6 Table Percentage of cells expressing checkpoint ligands in healthy donors and MDS patients. (PDF)

S7 Table Percentage of subpopulations with checkpoint receptors and ligands from total nucleated cells in bone marrow in PCA clusters with differences in survival. (PDF)

S1 Fig Multivariate Cox regression analysis of overall survival incorporating identified immunological clusters and IPSS-R. (PNG)
