# Supplementary material for: Landscape of alterations in the checkpoint system in myelodysplastic syndrome and implications for prognosis
Source: PLoS One. 2022 Oct 25;17(10):e0275399. doi: 10.1371/journal.pone.0275399 (PMC9595516; doi:10.1371/journal.pone.0275399)
Supplement: S1 Table — (PDF) [file pone.0275399.s001.pdf]

Supplementary table S1. Disposition of antibodies and fluorochromes

FITC	PE	PerCP	PE-Cy7	APC	APC-Cy7	VioBlue	VioGreen
T-cell and NK cell subpopulations							
CD3	CD8	CD4	CD279	CD152	CD278	CD223	CD45
CD3	CD8	CD4	CD56	TIM3	CD272	CD16	CD45
Expression of ligans on hematopoietic precursors							
HLA-DR	CD117	CD34	CD274	CD273	CD275	CD276	CD45
HLA-DR	CD80	CD34	CD279	CD152	CD117	CD86	CD45
Suppressor populations							
CD15	CD11b	CD14	CD274	CD273	CD272	CD276	CD45
CD127	CD25	CD4	CD274	CD273	CD272	CD276	CD45
