# Supplementary material for: Landscape of alterations in the checkpoint system in myelodysplastic syndrome and implications for prognosis
Source: PLoS One. 2022 Oct 25;17(10):e0275399. doi: 10.1371/journal.pone.0275399 (PMC9595516; doi:10.1371/journal.pone.0275399)
Supplement: S2 Table — (PDF) [file pone.0275399.s002.pdf]

Supplementary table S2. List of subpopulations analyzed

Subpopulation	Description
**total lymphocytes**	Total B and T lymphocytes
**CD3+**	total T lymphocytes
**CD3+CD8+**	Effector T lymphocytes
**CD3+CD4+**	Helper T lymphocytes
**CD4+CD8+**	Double-positive T cells
**CD3-CD56+**	NK cells
**CD3+CD56+**	NKT cells
**CD16+CD56-**	Subpopulation of NK cells with possible regulatory functions
**CD16+CD56+**	Cytotoxic subpopulation of NK cells
**CD16-CD56+**	Cytotoxic subpopulation of NK cells
**CD4+CD25+CD127low**	T-regulatory cells
**HLA-DRlow CD33+CD15+ CD11b+CD14-**	Myeloid-derived suppressor cells
**HLA-DRlow CD33+CD15- CD11b+CD14+**	Myeloid-derived suppressor cells
**CD117+CD34+HLA-DR+**	Bone marrow blast phenotype recommended for blast quantification in MDS
**CD117+CD34+HLA-DR-**	Hematopoietic precursors at the stage of maturation
**CD8+CD279+**	PD-1-positive effector T lymphocytes
**CD8+CD152+**	CTLA-4-positive effector T lymphocytes
**CD8+CD223+**	LAG-3-positive effector T lymphocytes
**CD8+TIM3+**	TIM-3-positive effector T lymphocytes
**CD4+CD279**	PD-1-positive effector T lymphocytes
**CD4+CD152**	CTLA-4-positive helper T lymphocytes
**CD4+CD223**	LAG-3-positive helper T lymphocytes
**CD4+TIM3+**	TIM-3-positive helper T lymphocytes
**CD3+CD279+**	PD-1-positive T lymphocytes
**CD3+CD152+**	CTLA-4-positive T lymphocytes
**CD3+CD223+**	LAG-3-positive T lymphocytes
**CD3 TIM3+**	TIM-3-positive T lymphocytes
**CD3-CD56+TIM3+**	General population of NK cells with TIM3 receptor
**CD16+CD56-TIM3+**	Subpopulation of NK cells with possible regulatory functions with TIM3 receptor
**CD16-CD56+TIM3+**	Cytotoxic subpopulation of NK cells with TIM3 receptor
**CD16+CD56+TIM3+**	Cytotoxic subpopulation of NK cells with TIM3 receptor
**CD117+CD34+HLA-DR+CD273+**	Bone marrow blasts expressing PD-1LG2 ligand
**CD117+CD34+HLA-DR+CD274+**	Bone marrow blasts expressing PD-1L ligand
**CD117+CD34+HLA-DR+CD275+**	Bone marrow blasts expressing B7-H2 ligand
**CD117+CD34+HLA-DR+CD152+**	Bone marrow blasts expressing B7-H3 ligand
**CD117+CD34+HLA-DR+CD80+**	Bone marrow blasts expressing B7-1 ligand
**CD117+CD34+HLA-DR+CD279+**	Bone marrow blasts expressing PD-1 receptor
**CD117+CD34+HLA-DR+TIM3+**	Bone marrow blasts expressing TIM-3 receptor
**CD117+CD34+HLA-DR-CD273+**	Immature blats with PD-1LG2 ligand
**CD117+CD34+HLA-DR-CD274+**	Immature blats with PD-1L ligand
**CD117+CD34+HLA-DR-CD275+**	Immature blats with B7-H2 ligand

**CD117+CD34+HLA-DR-CD152+**	Immature blats with B7-H3 ligand
**CD117+CD34+HLA-DR-CD80+**	Immature blats with B7-1 ligand
**CD117+CD34+HLA-DR-CD279+**	Immature blats with PD-1 receptor
**CD117CD34 HLA-DR-TIM3+**	Immature blats with TIM-3 receptor
**HLA-DR+CD273+**	General pool of antigen-presenting cells with PD-1LG2 ligand
**HLA-DR+CD274+**	General pool of antigen-presenting cells with PD-1L ligand
**HLA-DR+CD275+**	General pool of antigen-presenting cells with B7-H2 ligand
**HLA-DR+CD152+**	General pool of antigen-presenting cells with B7-H3 ligand
**HLA-DR+CD80+**	General pool of antigen-presenting cells with B7-1 ligand
**HLA-DR+CD279+**	General pool of antigen-presenting cells with PD-1 receptor
**HLA-DR+TIM3+**	General pool of antigen-presenting cells with TIM-3 receptor
**CD4+CD25+CD127low CD274+**	T-regulatory cells with PD-1L ligand
**CD4+CD25+CD127low CD273+**	T-regulatory cells with PD-1LG2 ligand
**CD8+CD278+**	Activated effector T cells with ICOS expression
**CD4+CD278+**	Activated helper T cells with ICOS expression
**CD3+CD278+**	Activated T cells with ICOS expression
**CD3-CD56CD278+**	Activated general pool of NK cells with ICOS expression
**CD16+CD56-CD278+**	Activated subpopulation of NK cells with ICOS expression
**CD16+CD56-CD272+**	Activated subpopulation of NK cells with BTLA expression
**CD16-CD56+CD278+**	Activated subpopulation of NK cells with ICOS expression
**CD16-CD56+CD272+**	Activated subpopulation of NK cells with BTLA expression
**CD117+CD34+HLA-DR+CD86+**	Bone marrow blasts expressing B7-2 ligand
**CD117+CD34+HLA-DR+CD276+**	Bone marrow blasts expressing B7-H3 ligand
**CD117+CD34+HLA-DR-Gal9+**	Immature blats with galectin-9 ligand
**CD16+CD56+CD278+**	Activated NKT cells with ICOS expression
**CD16+CD56+CD272+**	NKT cells with BTLA expression
**CD117+CD34+HLA-DR+Gal9+**	Bone marrow blasts with galectin-9 ligand
**CD117+CD34+HLA-DR-CD86+**	Immature blats with B7-2 ligand
**CD117+CD34+HLA-DR-CD276+**	Immature blats with B7-H3 ligand
**HLA-DR+gal9+**	General pool of antigen-presenting cells with galectin-9 ligand
**HLA-DR+CD86+**	General pool of antigen-presenting cells with B7-2 ligand
**HLA-DR+CD276+**	General pool of antigen-presenting cells with B7-H3 ligand
**HLA-DRlow CD33+CD15+ CD11b+CD14-CD274+**	Myeloid-derived suppressor cells expressing PD-1L ligand
**HLA-DRlow CD33+CD15+ CD11b+CD14-CD273**	Myeloid-derived suppressor cells expressing PD-1LG2 ligand
**HLA-DRlow CD33+CD15- CD11b+CD14-CD274**	Myeloid-derived suppressor cells expressing PD-1L ligand
**HLA-DRlow CD33+CD15- CD11b+CD14-CD273**	Myeloid-derived suppressor cells expressing PD-1LG2 ligand
**HLA-DRlow CD33-CD15+ CD11b+CD14-CD274+**	Myeloid-derived suppressor cells expressing PD-1L ligand
**HLA-DRlow CD33-CD15+ CD11b+CD14-CD273**	Myeloid-derived suppressor cells expressing PD-1LG2 ligand
