# Supplementary material for: Landscape of alterations in the checkpoint system in myelodysplastic syndrome and implications for prognosis
Source: PLoS One. 2022 Oct 25;17(10):e0275399. doi: 10.1371/journal.pone.0275399 (PMC9595516; doi:10.1371/journal.pone.0275399)
Supplement: S4 Table — (PDF) [file pone.0275399.s004.pdf]

Supplementary table S4. Percentage of cells expressing checkpoint receptors in healthy donors and MDS patients.

	MDS patients		Healthy donors	
Subopopulation	% of cells with receptor	SD% of cells with receptor	% of cells with receptor	SD% of cells with receptor
**% of CD8 with CD279**	58,23%	24,85%	41,17%	17,50%
**% of CD8 with CD152**	0,08%	0,10%	0,07%	0,08%
**% of CD8 with CD223**	0,27%	1,13%	0,01%	0,02%
**% of CD8 with TIM3**	4,71%	9,68%	1,68%	2,03%
**% of CD4 with CD279**	52,84%	27,37%	31,27%	20,17%
**% of CD4 with CD152**	0,20%	0,36%	0,07%	0,07%
**% of CD4 with CD223**	0,18%	0,52%	0,02%	0,03%
**% of CD4 with TIM3**	3,26%	9,43%	0,58%	0,24%
**% of CD3 with CD279**	55,35%	25,00%	33,36%	18,68%
**% of CD3 with CD152**	0,10%	0,14%	0,04%	0,03%
**% of CD3 with CD223**	0,21%	0,86%	0,01%	0,01%
**% of CD3 with TIM3**	3,95%	9,51%	0,62%	0,31%
**% of CD16CD56neg with Tim3**	33,01%	23,89%	11,60%	9,98%
**% of CD16negCD56 with Tim3**	13,16%	18,80%	14,86%	28,80%
**% of CD16CD56 with Tim3**	45,55%	27,63%	28,17%	24,30%
**% of CD8 with CD278**	2,71%	2,56%	1,85%	1,61%
**% of CD4 with CD278**	6,42%	5,25%	4,85%	2,52%
**% of CD3 with CD278**	4,30%	3,62%	2,61%	1,50%
**% of CD3negCD56 with CD278**	1,61%	3,26%	5,22%	8,17%
% of CD16CD56neg with CD278	3,61%	3,74%	3,20%	3,24%
**% of CD16CD56neg with CD272**	4,87%	5,02%	24,99%	44,89%
**% of CD16negCD56 with CD278**	2,42%	2,44%	3,80%	2,10%
**% of CD16negCD56 with CD272**	1,86%	1,72%	0,71%	0,52%
**% of CD16CD56 with CD278**	2,21%	4,11%	1,78%	1,04%
**% of CD16CD56 with CD272**	1,03%	1,18%	1,44%	1,47%
