# Supplementary material for: Landscape of alterations in the checkpoint system in myelodysplastic syndrome and implications for prognosis
Source: PLoS One. 2022 Oct 25;17(10):e0275399. doi: 10.1371/journal.pone.0275399 (PMC9595516; doi:10.1371/journal.pone.0275399)
Supplement: S5 Table — Differences determined in cluster analysis are discussed in the text. (PDF) [file pone.0275399.s005.pdf]

Supplementary table S5. Percentage of subpopulations with checkpoint ligands from total nucleated cells in bone marrow in healthy donors and MDS patients. Differences determined in cluster analysis are discussed in the text.

	**MDS patients**		**Healthy donors**	
**Subpopulation**	Mean % of NC	SD % of NC	Mean % of NC	SD % of NC
**CD117+CD34+HLA-DRlow**	3.952%	5.926%	0.532%	0.280%
**CD117+CD34+HLA-DR-**	0.506%	1.492%	0.011%	0.006%
**CD4+CD25+CD127low**	0.819%	0.632%	0.452%	0.175%
**HLA-DRlow CD33+CD15-CD11b+CD14+**	0.503%	0.840%	0.944%	0.831%
**HLA-DRlow CD33+CD15+ CD11b+CD14-**	2.170%	5.153%	0.211%	0.153%
**CD117+CD34+HLA-DRlowCD273+**	0.381%	3.413%	0.003%	0.003%
**CD117+CD34+HLA-DRlowCD274+**	1.264%	2.276%	0.062%	0.055%
**CD117+CD34+HLA-DRlowCD275+**	0.367%	2.746%	0.003%	0.001%
**CD117+CD34+HLA-DRlowCD80+**	0.030%	0.057%	0.088%	0.265%
**CD117+CD34+HLA-DRlowCD279+**	1.277%	2.575%	0.121%	0.082%
**CD117+CD34+HLA-DRlowTIM3+**	0.834%	3.881%	0.038%	0.035%
**CD117+CD34+HLA-DR-CD273+**	0.076%	0.578%	0.000%	0.000%
**CD117+CD34+HLA-DR-CD274+**	0.096%	0.433%	0.001%	0.001%
**CD117+CD34+HLA-DR-CD275+**	0.074%	0.492%	0.000%	0.000%
**CD117+CD34+HLA-DR-CD80+**	0.010%	0.038%	0.001%	0.002%
**CD117+CD34+HLA-DR-CD279+**	0.088%	0.445%	0.003%	0.002%
**CD117+CD34+HLA-DR-TIM3+**	0.035%	0.120%	0.002%	0.001%
**HLA-DR+CD273+**	1.006%	3.570%	0.192%	0.097%
**HLA-DR+CD274+**	5.754%	5.090%	3.244%	1.450%
**HLA-DR+CD275+**	0.575%	2.722%	0.127%	0.070%
**HLA-DR+CD80+**	1.061%	1.712%	0.794%	1.264%
**HLA-DR+CD279+**	7.386%	5.798%	8.245%	12.800%
**HLA-DR+TIM3+**	4.986%	7.397%	3.260%	1.331%
**CD4+CD25+CD127low CD274+**	0.043%	0.102%	0.039%	0.117%
**CD4+CD25+CD127low CD273+**	0.003%	0.005%	0.000%	0.001%
**CD4+CD25+CD127low CD223+**	0.002%	0.005%	0.000%	0.000%
**CD117+CD34+HLA-DRlowGal9+**	0.014%	0.021%	0.000%	0.000%
**CD117+CD34+HLA-DRlowCD276+**	0.026%	0.071%	0.000%	0.000%
**CD117+CD34+HLA-DRlowCD86+**	0.599%	4.344%	0.000%	0.000%
**CD117+CD34+HLA-DR-Gal9+**	0.077%	0.418%	0.009%	0.022%
**CD117+CD34+HLA-DR-CD276+**	0.000%	0.001%	0.000%	0.000%
**CD117+CD34+HLA-DR-CD86+**	0.001%	0.003%	0.000%	0.000%
**HLA-DR+ Gal9+**	0.890%	1.981%	0.530%	0.400%
**HLA-DR+ CD276+**	0.739%	1.578%	0.000%	0.000%
**HLA-DR+ CD86+**	0.456%	0.530%	0.000%	0.000%
**HLA-DRlow CD33+CD15+CD11b+CD14- CD274+**	0.136%	0.215%	0.067%	0.104%
**HLA-DRlow CD33+CD15+CD11b+CD14- CD273+**	0.051%	0.168%	0.023%	0.022%
**HLA-DRlow CD33+CD15-CD11b+CD14+ CD274+**	0.273%	0.471%	0.561%	0.602%
**HLA-DRlow CD33+CD15-CD11b+CD14+ CD273+**	0.007%	0.016%	0.006%	0.007%
