# Supplementary material for: Landscape of alterations in the checkpoint system in myelodysplastic syndrome and implications for prognosis
Source: PLoS One. 2022 Oct 25;17(10):e0275399. doi: 10.1371/journal.pone.0275399 (PMC9595516; doi:10.1371/journal.pone.0275399)
Supplement: S6 Table — (PDF) [file pone.0275399.s006.pdf]

Supplementary table S6. Percentage of cells expressing checkpoint ligands in healthy donors and MDS patients.

	**MDS patients**		**Healthy donors**	
**Subpopulation**	% of cells with ligand	SD% of cells with ligand	% of cells with ligand	SD% of cells with ligand
**% of CD117+CD34+HLA-DRlow with CD273+**	2,57%	10,14%	0,57%	0,41%
**% of CD117+CD34+HLA-DRlow with CD274+**	31,06%	23,28%	12,04%	7,48%
**% of CD117+CD34+HLA-DRlow with CD275+**	3,24%	9,16%	0,63%	0,46%
**% of CD117+CD34+HLA-DRlow with CD80+**	2,31%	5,74%	9,14%	25,64%
**% of CD117+CD34+HLA-DRlow with CD279+**	31,92%	25,58%	28,60%	18,21%
**% of CD117+CD34+HLA-DRlow with TIM3+**	15,34%	24,00%	6,35%	3,21%
**% of CD117+CD34+HLA-DR- with CD273+**	5,66%	17,29%	1,78%	2,17%
**% of CD117+CD34+HLA-DR- with CD274+**	21,90%	23,18%	10,73%	6,81%
**% of CD117+CD34+HLA-DR- with CD275+**	5,00%	15,63%	1,01%	1,52%
**% of CD117+CD34+HLA-DR- with CD80+**	6,60%	12,23%	14,73%	18,48%
**% of CD117+CD34+HLA-DR- with CD279+**	18,27%	29,67%	32,38%	20,64%
**% of CD117+CD34+HLA-DR- with TIM3+**	7,19%	12,46%	18,81%	18,16%
**% of CD4+CD25+CD127low with CD274+**	5,19%	12,00%	6,70%	19,21%
**% of CD4+CD25+CD127low with CD273+**	0,45%	0,74%	0,11%	0,18%
**% of CD4+CD25+CD127low with CD223+**	0,14%	0,62%	0,03%	0,06%
**% of HLA-DRlow CD33+CD15+CD11b+CD14- with CD274+**	7,67%	18,55%	3,17%	5,85%
**% of HLA-DRlow CD33+CD15+CD11b+CD14- with CD273+**	2,03%	5,23%	1,02%	1,07%
**% of HLA-DRlow CD33+CD15-CD11b+CD14+ with CD274+**	6,28%	21,00%	23,02%	24,90%
**% of HLA-DRlow CD33+CD15-CD11b+CD14+ with CD273+**	0,27%	0,75%	0,25%	0,31%
