# Supplementary material for: Landscape of alterations in the checkpoint system in myelodysplastic syndrome and implications for prognosis
Source: PLoS One. 2022 Oct 25;17(10):e0275399. doi: 10.1371/journal.pone.0275399 (PMC9595516; doi:10.1371/journal.pone.0275399)
Supplement: S7 Table — (PDF) [file pone.0275399.s007.pdf]

Supplementary table S7. Percentage of subpopulations with checkpoint receptors and ligands from total nucleated cells in bone marrow in PCA clusters with differences in survival.

	Cluster 1		Cluster 2		Cluster 3	
**Subpopulation**	Mean % of NC	SD % of NC	Mean % of NC	SD % of NC		
**total lymphocytes**	24.45%	17.16%	21.16%	15.50%	24.07%	19.38%
**CD3+**	18.49%	14.22%	16.07%	11.26%	17.65%	14.11%
**CD3+CD8+**	7.92%	6.81%	7.28%	5.81%	7.87%	7.25%
**CD3+CD4+**	9.66%	7.52%	8.00%	6.19%	8.80%	7.05%
**CD4+CD8+**	0.20%	0.31%	0.16%	0.23%	0.31%	0.63%
**CD3-CD56+**	2.35%	2.50%	3.33%	3.95%	3.88%	5.56%
**CD3+CD56+**	1.55%	1.64%	2.54%	5.55%	2.54%	3.07%
**CD16+CD56-**	0.61%	0.87%	0.65%	0.60%	0.36%	0.47%
**CD16+CD56+**	2.25%	2.37%	2.84%	3.67%	3.59%	5.56%
**CD16-CD56+**	1.81%	1.85%	2.22%	2.02%	2.74%	2.96%
**CD4+CD25+CD127low**	0.89%	0.69%	0.65%	0.45%	0.80%	0.67%
**HLA-DRlow CD33+CD15-CD11b+CD14+**	0.56%	0.71%	0.75%	1.11%	0.31%	0.43%
**HLA-DRlow CD33+CD15+CD11b+CD14-**	2.38%	7.09%	1.39%	2.16%	1.60%	2.18%
**CD8+CD279+**	0.00%	0.01%	0.03%	0.13%	0.01%	0.02%
**CD8+CD152+**	0.08%	0.08%	0.65%	1.39%	0.19%	0.19%
**CD8+CD223+**	5.31%	6.62%	3.77%	3.35%	4.11%	3.45%
**CD8+TIM3+**	0.01%	0.01%	0.03%	0.07%	0.03%	0.07%
**CD4+CD279**	0.01%	0.01%	0.01%	0.02%	0.00%	0.01%
**CD4+CD152**	0.20%	0.68%	0.69%	3.09%	0.07%	0.05%
**CD4+CD223**	9.88%	11.37%	8.21%	6.99%	8.93%	6.36%
**CD4+TIM3+**	0.01%	0.01%	0.02%	0.05%	0.02%	0.05%
**CD3+CD279+**	0.01%	0.02%	0.04%	0.14%	0.01%	0.02%
**CD3+CD152+**	0.29%	0.69%	1.22%	3.92%	0.24%	0.17%
**CD3+CD223+**	0.56%	0.92%	1.88%	2.99%	1.24%	1.55%
**CD3 TIM3+**	0.13%	0.33%	0.26%	0.36%	0.11%	0.19%
**CD3-CD56+TIM3+**	0.04%	0.02%	0.31%	0.23%	0.18%	0.19%
**CD16+CD56-TIM3+**	0.53%	0.85%	1.64%	2.86%	1.18%	1.54%
**CD16-CD56+TIM3+**	0.18%	0.25%	0.24%	0.30%	0.25%	0.31%
**CD16+CD56+TIM3+**	0.66%	0.91%	0.57%	0.42%	0.51%	0.64%
**CD8+CD278+**	0.77%	0.99%	0.77%	0.59%	0.74%	0.92%
**CD4+CD278+**	0.11%	0.14%	0.38%	0.54%	0.16%	0.17%
**CD3+CD278+**	0.01%	0.02%	0.02%	0.02%	0.04%	0.06%
**CD3-CD56CD278+**	0.40%	0.28%	0.58%	0.67%	0.82%	1.08%
**CD16+CD56-CD278+**	0.69%	0.52%	1.11%	1.17%	1.66%	2.13%
**CD16+CD56-CD272+**	0.03%	0.04%	0.04%	0.06%	0.04%	0.07%
**CD16-CD56+CD278+**	0.01%	0.01%	0.03%	0.04%	0.00%	0.00%
**CD16-CD56+CD272+**	0.01%	0.01%	0.03%	0.02%	0.03%	0.04%
**CD117+CD34+HLA-DRlow**	0.88%	0.97%	0.97%	0.96%	10.20%	7.07%
**CD117+CD34+HLA-DR-**	0.07%	0.13%	0.14%	0.62%	1.32%	2.36%
**CD4+CD25+CD127low**	0.89%	0.69%	0.65%	0.45%	0.80%	0.67%
**HLA-DRlow CD33+CD15-CD11b+CD14+**	0.56%	0.71%	0.75%	1.11%	0.31%	0.43%
**HLA-DRlow CD33+CD15+CD11b+CD14-**	2.38%	7.09%	1.39%	2.16%	1.60%	2.18%
**CD117+CD34+HLA-DRlowCD273+**	0.01%	0.01%	0.01%	0.01%	1.17%	6.01%
**CD117+CD34+HLA-DRlowCD274+**	0.22%	0.38%	0.33%	0.52%	3.26%	3.14%
**CD117+CD34+HLA-DRlowCD275+**	0.01%	0.02%	0.01%	0.02%	1.11%	4.81%
**CD117+CD34+HLA-DRlowCD80+**	0.04%	0.15%	0.01%	0.01%	0.07%	0.09%
**CD117+CD34+HLA-DRlowCD279+**	0.21%	0.31%	0.30%	0.53%	3.43%	3.73%

**CD117+CD34+HLA-DRlowTIM3+**	0.12%	0.30%	0.06%	0.07%	2.10%	6.32%
**CD117+CD34+HLA-DR-CD273+**	0.00%	0.00%	0.00%	0.00%	0.23%	1.01%
**CD117+CD34+HLA-DR-CD274+**	0.01%	0.01%	0.07%	0.35%	0.20%	0.65%
**CD117+CD34+HLA-DR-CD275+**	0.00%	0.00%	0.01%	0.07%	0.21%	0.86%
**CD117+CD34+HLA-DR-CD80+**	0.00%	0.00%	0.00%	0.01%	0.03%	0.07%
**CD117+CD34+HLA-DR-CD279+**	0.01%	0.02%	0.07%	0.35%	0.18%	0.68%
**CD117+CD34+HLA-DR-TIM3+**	0.01%	0.02%	0.01%	0.02%	0.08%	0.19%
**HLA-DR+CD273+**	0.42%	0.43%	0.76%	1.03%	1.74%	6.18%
**HLA-DR+CD274+**	3.51%	2.53%	5.24%	4.37%	8.23%	6.38%
**HLA-DR+CD275+**	0.21%	0.29%	0.30%	0.50%	1.20%	4.75%
**HLA-DR+CD80+**	0.45%	0.49%	0.36%	0.59%	1.07%	2.39%
**HLA-DR+CD279+**	0.89%	1.28%	0.90%	0.94%	1.37%	2.54%
**HLA-DR+TIM3+**	5.75%	7.28%	7.40%	6.14%	9.66%	6.42%
**CD4+CD25+CD127low CD274+**	3.78%	6.59%	4.43%	4.77%	6.16%	8.70%
**CD4+CD25+CD127low CD273+**	0.05%	0.12%	0.04%	0.12%	0.03%	0.04%
**CD4+CD25+CD127low CD223+**	0.00%	0.01%	0.00%	0.00%	0.00%	0.00%
**CD117+CD34+HLA-DRlowGal9+**	0.00%	0.01%	0.00%	0.01%	0.00%	0.00%
**CD117+CD34+HLA-DRlowCD276+**	0.02%	0.03%	0.01%	0.02%	0.01%	0.01%
**CD117+CD34+HLA-DRlowCD86+**	0.02%	0.04%	0.03%	0.10%	0.02%	0.03%
**CD117+CD34+HLA-DR-Gal9+**	0.00%	0.00%	0.00%	0.00%	1.67%	7.26%
**CD117+CD34+HLA-DR-CD276+**	0.01%	0.02%	0.01%	0.02%	0.20%	0.69%
**CD117+CD34+HLA-DR-CD86+**	0.00%	0.00%	0.00%	0.00%	0.00%	0.00%
**HLA-DR+ Gal9+**	0.00%	0.00%	0.00%	0.00%	0.00%	0.00%
**HLA-DR+ CD276+**	0.62%	0.54%	0.92%	2.05%	1.01%	2.57%
**HLA-DR+ CD86+**	0.29%	0.39%	1.06%	2.27%	0.73%	0.98%
**HLA-DRlow CD33+CD15+CD11b+CD14-CD274+**	0.54%	0.60%	0.49%	0.58%	0.31%	0.36%
**HLA-DRlow CD33+CD15+CD11b+CD14-CD273+**	0.12%	0.19%	0.09%	0.13%	0.17%	0.27%
**HLA-DRlow CD33+CD15-CD11b+CD14+ CD274+**	0.07%	0.23%	0.03%	0.05%	0.04%	0.09%
**HLA-DRlow CD33+CD15-CD11b+CD14+ CD273+**	0.38%	0.48%	0.26%	0.52%	0.33%	0.53%
