# Supplementary figures and images for: Landscape of alterations in the checkpoint system in myelodysplastic syndrome and implications for prognosis
Source: PLoS One. 2022 Oct 25;17(10):e0275399. doi: 10.1371/journal.pone.0275399 (PMC9595516; doi:10.1371/journal.pone.0275399)

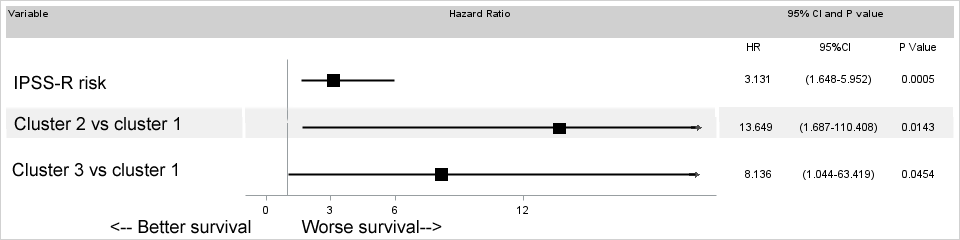

Supplement: S1 Fig — (PNG) [file pone.0275399.s008.png]
